# Public health response to an outbreak of SARS-CoV2 infection in a Barcelona prison

**DOI:** 10.1017/S0950268821000789

**Published:** 2021-04-14

**Authors:** A. Marco, C. Gallego, V. Pérez-Cáceres, R. A. Guerrero, M. Sánchez-Roig, R. M. Sala-Farré, J. Fernández-Náger, E. Turu

**Affiliations:** 1 Prison Health Program, Catalan Institute of Health, Barcelona, Spain; 2 CIBER Epidemiología y Salud Pública (CIBERESP), Madrid, Spain; 3 Primary Penitentiary Care Team, La Roca del Vallés 1, Barcelona, Spain; 4 Epidemiological Surveillance Service and Response to Public Health Emergencies. Vallés Occidental and Vallés Oriental, Barcelona, Spain

**Keywords:** COVID-19, infectious disease, outbreak, patient isolation, prisons, public health

## Abstract

An outbreak of SARS-CoV2 infection in a Barcelona prison was studied. One hundred and forty-eight inmates and 36 prison staff were evaluated by rt-PCR, and 24.1% (40 prisoners, two health workers and four non-health workers) tested positive. In all, 94.8% of cases were asymptomatic. The inmates were isolated in prison module 4, which was converted into an emergency COVID unit. There were no deaths. Generalised screening and the isolation and evaluation of the people infected were key measures. Symptom-based surveillance must be supplemented by rapid contact-based monitoring in order to avoid asymptomatic spread among prisoners and the community at large.

## Background

On 14 March, a state of emergency was declared in Spain due to the spread of the SARS-CoV-2 infection. In prisons, mobility and interpersonal contacts were severely restricted, with the suspension or reduction of prison activities, communications and permits. A 14-day confinement was also ordered for new admissions. These restrictions were approved by a Ministerial Order. The government of Catalonia (an autonomous community in Spain) has responsibility for health and prison policy throughout the region. In Catalonia, 8300 inmates are held in nine prisons and in five open penitentiary centres. Prison medical services, health programs and healthcare circuits depend on the public health system managed by the Catalan Institute of Health. Up until mid-April, in order to reduce the population exposed, the Catalan government released 17% of the prison population (*n* = 1425 inmates). This figure was considerably higher than the average of the countries of the European Union, which was 5.1% [1]. Seventeen days after adopting these measures, when 13 cases had been diagnosed in four other prisons, the first cases of SARS-CoV2 Infection were detected in Quatre Camins Prison (henceforth, QCP).

## Outbreak detection

QCP is located in La Roca del Vallés, in the province of Barcelona. It houses 946 inmates, all male, in 14 residence modules plus an extra module for new entrants, and a nursing department. It is a prison for inmates who have already been sentenced, but presents no other differences from the rest of the prisons managed by the Government of Catalonia.

Between 31 March and 9 April, QCP reported seven cases in inmates in module 4 (MR4) of the prison. For this reason, the decision was taken to study all the inmates admitted to QCP MR4, as well as the health workers and non-health workers who had had close contact with this population group. Family members were not studied because there had been no contact with people from outside the prison since 15 March.

For the study of the outbreak, ‘close contacts’ were identified, using the definition of the Spanish Ministry of Health: that is, in the prison context, people who had had contact with the case in the 48 h before the onset of symptoms (or diagnosis, in the case of asymptomatics) until the moment when the case was isolated.

### Screening strategy and case selection

On 9 April 2020, all MR4 inmates and the staff who had been in close contact with them in the past 14 days were administered the real-time reverse transcription polymerase chain reaction test (rt-PCR) with samples of nasopharyngeal/oropharyngeal exudate. All the samples were analysed at the laboratory of the Germans Trias University Hospital and were categorised into two groups: (a) symptomatic cases or (b) close non-symptomatic contacts.

Although the care of health workers is also managed by the Catalan Institute of Health, it is overseen by a different department and access to health workers' data is restricted. For this reason, the rt-PCR was performed in health workers who might have had contact with MR4 inmates, but if the infection was confirmed, the worker was referred for control and follow-up by his/her own healthcare network. Pending the results of the rt-PCR, the inmates were left in isolation in MR4.

## Findings

One hundred eighty-four subjects were screened: (a) 148 inmates (145 inmates in MR4 and three more from that module but who were in isolation in the Nursing Department due to their previous close contact with inmates diagnosed with the infection); (b) 31 non-health workers; and (c) five health workers: one doctor, one nurse and three health assistants. There were no significant differences (*P* = 0.39) between the patients screened and the rest of the prison population in terms of age distribution, origin and comorbidity. One hundred forty-six of the inmates screened were asymptomatic and two had mild symptoms (one case with ageusia and anosmia and another with low fever and general discomfort). Only one of the health workers presented clinical symptoms (fever, cough and moderate respiratory distress). All non-health workers were asymptomatic.

**Fig. 1.**
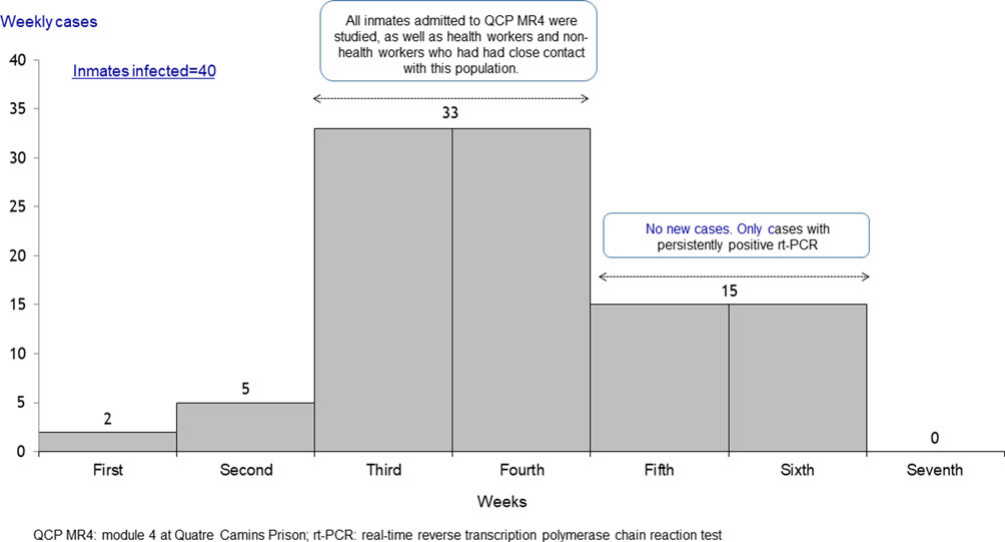
Epicurve of the SARS-CoV-2 infection outbreak in inmates in module 4 of the Quatre Camins Prison.

**Fig. 2.**
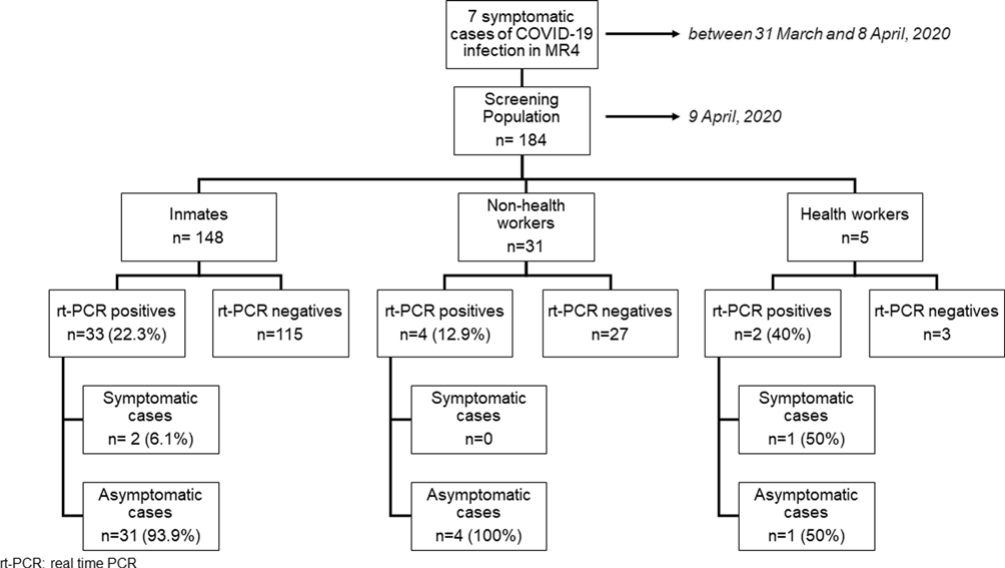
Distribution according to population group, rt-PCR result and presence/absence of symptoms.

In addition to the seven cases already diagnosed, 39 more were positive on rt-PCR screening: three symptomatic and 36 asymptomatic subjects. The positive rt-PCR rate was therefore 24.1%. Figure 1 shows the epicurve of the outbreak. Figure 2 shows the distribution according to the group studied, the rt-PCR results and the presence/absence of symptoms.

Regarding clinical evolution, only two individuals (one inmate and one health worker), were admitted to the hospital, but neither required intensive care. All 39 patients had an uneventful recovery.

**Fig. 3.**
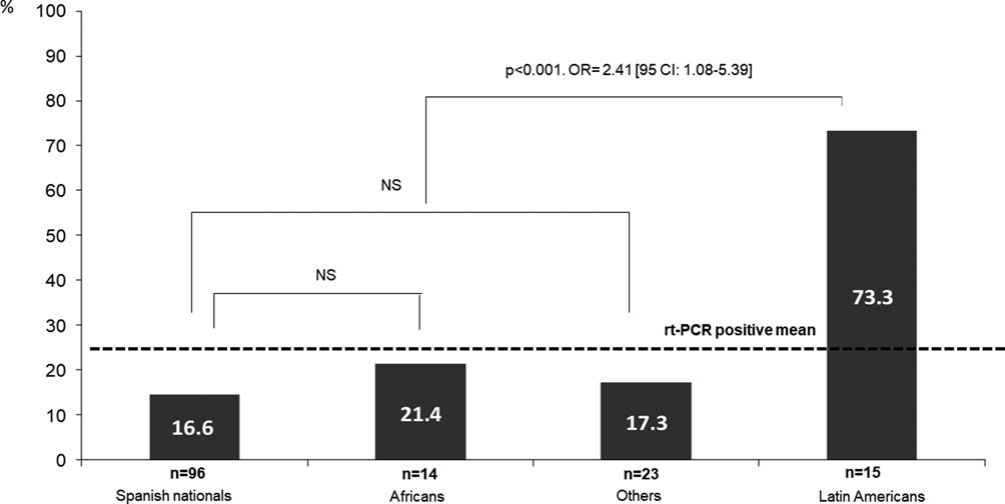
Distribution of positive rt-PCR according to the origin of the studied patients.

All inmates were men with a mean age of 40 ± 7.3 years (range 21–76 years). Seven (4.7%) were ≥60 years old and 52 (35.1%) were not Spanish. Thirteen (8.8%) were infected with HIV, but all presented adequate virological and immunological control. There were no statistically significant differences between inmates with positive or negative rt-PCR in terms of age, history of diabetes, HIV infection or HCV infection (Table 1). According to origin, however, significant differences were found, since more people of Latin American, origin had positive rt-PCR (73.3% *vs.* 16.5% in those of other origins; *P* < 0.001; OR 2.41, 95% CI 1.08–5.39). Figure 3 shows the proportion of positive rt-PCR according to the origin of the patients.

**Table 1.** Descriptive characteristics of the inmates screened according to rt-PCR result

Variable	Scanned population, *n* = 148	Cases with positive rt-PCR, *n* = 33 (%)	Cases with negative rt-PCR, *n* = 115 (%)	*P* value
Mean age	39.7	39.9	39.2	0.91
Spanish national				0.01
Yes	96 (64.9)	16 (16.7)	80 (83.3)	
No	52 (35.1)	17 (32.7)	35 (67.3)	
History of diabetes				0.40
Yes	8 (5.4)	2 (25.0)	6 (75.0)	
No	140 (94.6)	31 (22.1)	109 (77.9)	
HIV infection				0.45
Yes	13 (8.8)	3 (23.1)	10 (76.9)	
No	135 (91.2)	30 (22.2)	105 (77.8)	
HCV infection				0.30
Yes	2	0 (0)	2 (100)	
No	146	33 (22.6)	113 (77.4)	

rt-PCR, real-time PCR.

## Outbreak control measures

Isolation at home was recommended in the six workers with positive rt-PCR tests, and the result was reported to the Occupational Risk Prevention Unit. The subsequent control and follow-up were carried out by their corresponding medical services. As for inmates, those with positive rt-PCR were isolated in MR4, which was disinfected sequentially by zones. Inmates with negative rt-PCR were confined in MR1.

MR4 was considered an emergency COVID-19 unit, created because of the number of infected inmates who were asymptomatic or mildly symptomatic but did not present criteria for hospitalisation. The unit adopted a series of organisational and functional measures to guarantee the safety, quality and efficiency of the care given to these low complexity cases. Cleaning, laundry, waste management and the distribution of food and medication were organised according to the recommendations of the Catalan Health Service [2].

The following controls were imposed: (a) strict isolation of the unit, which only key health and non-health workers were authorised to enter and leave; (b) use of individual protection equipment; and (c) clinical controls (oxygen saturation, temperature and enquiries about the appearance of symptoms) twice daily. During the stay in isolation, only one of the 33 inmates admitted was hospitalised (due to clinical deterioration).

The inmates with negative rt-PCR were transferred to MR1, where they were placed in confinement. There they were allowed to share some spaces in small groups, but wearing a mask at all times. The aim was to ensure that they were not incubating the SARS-CoV2 infection or were rt-PCR ‘false negatives’. Therefore, in this a situation of confinement clinical controls (oxygen saturation, temperature and enquiries about the appearance of suspicious symptoms) was performed twice daily. No inmate presented fever or any clinical suspicion of COVID-19 during the period of confinement.

Just over half (51.5%) presented a negative rt-PCR after 14 days of isolation, and 81.8% at 21 days, while 18.2% were negative only after 4 weeks.

## Discussion

This report of the outbreak at the QCP is one of the first descriptions (if not *the* first) of COVID-19 in the prison setting in Europe. SARS-CoV2 infection was detected in 40 inmates and six workers (24.1%) of the individuals studied. This rate is high, though below the 30% observed in a long-term care nursing centre [3], and below the 35% recorded in an outbreak in a hospital [4] (although the latter report corresponded to the first indigenous case of COVID-19 infection in the USA, so it was unsuspected and exposure was increased by the use of multiple aerosol generation procedures).

The rate reported in this outbreak is higher than the figure of 20.7% observed in the Diamond Princess Cruise passengers after 14 days of quarantine in February 2020 [5]. It has been calculated that in the cruise ship, the R0 (the mean number of people who will contract a disease from one contagious person) was 5–14 times higher than the normal figure of 1.5–3.0, because of the high occupant density and confined space [6]. A similar process, even more intense, may have occurred in the QCP MR4. SARS-CoV2 spreads widely in closed spaces and this is probably the reason why 24.1% of the admissions in MR4, and 78.6% of the Latin American inmates, became infected. In prisons, members of racial and ethnic minorities tend to stick together and protect each other, and share cells, activities and even food. There is no greater genetic predisposition to infection in ethnic or racial groups. Therefore, the expansion of the infection and the very high positive rt-PCR rate in the Latin American inmates were presumably due to the close contact they maintain with each other.

It was not possible to identify the index case. In this infection, the mean incubation time is 5.1 days, but 97.5% of symptomatic cases occur within 11.5 days of exposure [7], so the index case may have been asymptomatic or one of the initial seven cases. The lack of cases prior to the outbreak and restrictions regarding contact with the outside world (no prison leave, no visits and 14-day confinement of new admissions) suggest that the transmission may have started from an asymptomatic case, either a prisoner or (more likely) a member of the prison or health staff.

In all, 94.8% cases were asymptomatic. These cases are more difficult to detect, because they do not arouse suspicion and they are either not diagnosed at all or underdiagnosed. In addition, as these people do not feel ill, they continue to interact as normal with others and thus transmit the infection [8]. In fact, it has been estimated that silent disease transmission during the presymptomatic and asymptomatic stages is responsible for more than 50% of the overall attack rate in COVID-19 outbreaks [9].

Regarding age, the subjects affected were young (mean age 40 years) and many had no relevant medical history. As is customary in these patients, the evolution was satisfactory and no deaths were recorded. The case fatality rate of zero is a very important finding and needs to be emphasised. In addition to the younger age of the patients, the highly effective measures adopted probably had a bearing on the results.

It should be remembered that 9.1% of the inmates infected in the outbreak had HIV infection. There were no differences in the rt-PCR result according to HIV status, suggesting that people with HIV are not more prone to SARS-CoV-2 infection. It should be noted that all were virologically controlled and had a CD4/mm3 lymphocyte count greater than 200. Currently, there is no solid data to show that HIV-infected individuals with COVID-19 present poorer clinical outcomes course if they are well controlled and exhibit a moderate immunity [10].

It should also be noted that 94.8% of subjects with positive rt-PCR did not present symptoms. One review estimated that between 40% and 45% of the cases of SARS-CoV-2 infection are asymptomatic [11]. As recently suggested, asymptomatic transmission is probably the Achilles heel of the pandemic [12]. Asymptomatic patients transmit the infection silently and may interact more with other people because they do not feel sick [8, 9]. In accordance with the reports of other outbreaks [13, 14], our results confirm that the isolation of both symptomatic and non-symptomatic patients and the study of all contacts is essential in order to control the outbreak.

As regards the control of outbreak, to manage we received the support of the prison authorities. This backing was vital in an environment that already poses notable organisational challenges, and in which other specific features (security, e.g.) add to the management difficulties.

In short, the outbreak presented here shows that outbreaks of SARS-CoV2 infection in closed environments are a real possibility. They pose a huge risk, must be detected early, are difficult to manage, and require optimal coordination between the health and prison authorities. When they occur, general screening by means of PCR and the isolation and evaluation of those infected are key measures. Symptom-based surveillance must be supplemented by rapid contact-based monitoring in order to avoid asymptomatic spread among prisoners, health workers and ultimately in the community at large.

## Data

The data that support the findings of this study are available from Prison Health Program (Catalan Institute of Health). Restrictions apply to the availability of these data as they are from the inmates’ medical records, which were used under license for this study. Data are available and can be requested from Dr Andrés Marco (amarco@gencat.cat) with prior authorisation from the Prison Health Program of the Catalan Health Institute.
